# Interactions among the Predatory Midge *Aphidoletes aphidimyza* (Diptera: Cecidomyiidae), the Fungal Pathogen *Metarhizium brunneum* (Ascomycota: Hypocreales), and Maize-Infesting Aphids in Greenhouse Mesocosms

**DOI:** 10.3390/insects8020044

**Published:** 2017-04-12

**Authors:** Ana Gorete Campos de Azevedo, Bernhardt Michael Steinwender, Jørgen Eilenberg, Lene Sigsgaard

**Affiliations:** Department of Plant and Environmental Science, University of Copenhagen, 1871 Frederiksberg C, Denmark; agca@plen.ku.dk (A.G.C.d.A.); b.steinwender@t-online.de (B.M.S.); jei@plen.ku.dk (J.E.)

**Keywords:** natural enemies, non-target-effects, pathogen, predator, *Aphidoletes aphidimyza*, *Metarhizium brunneum*

## Abstract

The generalist entomopathogenic fungus, *Metarhizium brunneum*, has proved to have great potential as a versatile biological pest control agent. The gall midge *Aphidoletes aphidimyza* is a specialist predator that occurs naturally in Europe and has been successfully used for aphid suppression. However, the interaction between these two biological control organisms and how it may affect the biological control of aphids awaits further investigation. As part of the EU-supported project INBIOSOIL, this study was conducted in greenhouse conditions to assess the possible effects of combining both biological control agents. In a randomized complete block design, sweet corn (*Zea mays* var. *saccharata*) plants were grown in large pots filled with natural soil or natural soil inoculated with *M. brunneum*. At the third leaf stage, before being individually caged, plants were infested with *Rhopalosiphum padi* and *A. aphidimyza* pupae were introduced in the soil. *Aphidoletes aphidimyza* midge emergence, number of living midges and number of aphids were recorded daily. The presence of conidia in the soil and on leaves was assessed during the experiment. At the conclusion of the experiment, the number of live aphids and their developmental stage, consumed aphids, and *A. aphidimyza* eggs was assessed under stereomicroscope. This study’s findings showed that the presence of *M. brunneum* did not affect *A. aphidimyza* midge emergence. However, longevity was significantly affected. As the study progressed, significantly fewer predatory midges were found in cages treated with *M. brunneum* compared to untreated cages. Furthermore, by the end of the study, the number of predatory midges found in the *Metarhizium*-treated cages was four times lower than in the untreated cages. Both daily and final count of aphids were significantly affected by treatment. *Aphidoletes aphidimyza* applied alone suppressed the aphid population more effectively than *M. brunneum* applied alone. Additionally, the aphid population was most suppressed when both agents were combined, though the suppression was less than additive.

## 1. Introduction

Aphids (Hemiptera: Aphididae) are phloem-feeding insects that affect a variety of crops in agriculture including maize, sorghum, wheat and barley [1]. An extensive range of natural enemies, such as entomopathogenic fungi and aphidophagous predators, attacks the aphid community, and interactions among these natural enemies can be expected [2]. *Rhopalosiphum padi* (L.) is one of the most important cereal pests in Denmark and Northern Europe; therefore, using biological control to suppress the aphid population could bring a decrease in pesticide use [3].

Several studies have demonstrated the ability of the entomopathogenic fungi from the genus *Metarhizium* to suppress aphid populations [4,5,6]. Additionally, *Metarhizium* has been found to cause mycoses in an important soil dwelling pest in maize, *Diabrotica virgifera* (Col., Chrysomelidae) [7], and it shows persistence in maize fields [8]. *Metarhizium brunneum* GranMet/BIPESCO 5 (Samen Schwarzenberger, Austria), the same genotype as used in this study, is the only strain registered commercially for using against pests in several European countries, officially deposited as ARSEF1095 or DSM3884 [9]. Although there have been studies concerning the non-target effects of *Metarhizium* on beneficial insects, few have dealt with these effects in more realistic conditions. Most laboratory studies show that beneficial insect populations face low risk from *Metarhizium* exposure [10,11,12]. However, the consequences of non-target effects on the success of biological control cannot be neglected and should be investigated more thoroughly.

*Aphidoletes aphidimyza* (Rondani) is an aphidophagous gall midge that occurs naturally in Europe and has been successfully used in biological control programs against a wide range of aphid species [13]. Females lay eggs on leaves close to aphid colonies; only the larvae are predaceous and crawl on the leaf surface searching for aphid prey. Each larva may consume 3–50 aphids per day, feeding by biting the leg of the aphid and paralyzing them with a toxin before sucking out the body fluids [14].

The effects of combining insect pathogens and arthropod natural enemies for biological control are of high relevance in many crop systems, as complex interactions may occur and impact the final outcome. The release of a biocontrol agent can affect the naturally occurring beneficial insects, causing direct or indirect effects on non-target species. Hence the introduction of multiple agents should be conducted with caution as different types of natural enemies can synergistically control herbivore populations, or they can negatively impact each other [15]. Many studies have investigated non-target effects of fungal pathogens on beneficial insects affecting aphid suppression [16,17,18]. However, there is a need to assess the non-target effects of fungal pathogens on *A. aphidimyza* because the focus has mainly been on *A. Aphidimyza* interacting with other arthropod natural enemies and nematodes [19,20,21].

As part of the EU FP7 project INBIOSOIL, this study was designed to evaluate the impact of soil applied *M. brunneum* on *A. aphidimyza* and the resulting suppression of maize-infesting *R. padi*. The predator pupates in the soil and thus could be directly exposed to *M. brunneum*, so it was hypothesized that the fungus could negatively affects the predator, as shown by [22]. It was further hypothesized that natural enemies from two remote functional groups would be complementary in terms of aphid population suppression, as previously found in studies with predators from different functional groups [23].

Considering that multitrophic effects originating from the host plant may greatly affect the results of the trial [24], this setup was developed to provide sufficient scale and complexity for expression of a wide range of potential interspecific interactions and a realistic measure of resulting pest population performance.

## 2. Materials and Methods

### 2.1. Source and Maintenance of Insects

Cohort rearing of *A. aphidimyza* was carried out by EWH BioProduction ApS (Tappernøje, Denmark) and maintained at 23 ± 0.5 °C, 50%–75% relative humidity and L16: D8 light regime. The company also has a mass-production of this species, complying with the IOBC quality control guidelines for beneficial arthropods [25]. For production of cohorts of *A. aphidimyza*, males and females were released into a cage (40 cm × 40 cm × 40 cm) with a pepper plant, infested with *Myzus persicae*. After 24 h, adults were removed and a whole pepper plant with *A. aphidimyza* eggs was transferred into a tray (10 cm × 10 cm × 5 cm) and covered with sand. The gall midge larvae emerging from eggs were fed daily with 10 mL of aphids (*Megoura* sp.) until pupation, which occurs in the sand [26]. For this experiment, 5–6 days old *A. aphidimyza* pupae were used.

*Rhopalosiphum padi* was provided on barley banker plants from the same company and maintained in a ventilated plexiglass cage (0.60 m × 0.30 m × 0.30 m) for one week at 21 °C until used in the experiment. Pilot tests showed that *R. padi* performed well after being transferred from barley to maize. Only apterous, virginoparous females were used in the experiment.

### 2.2. Source and Preparation of the Microbial Inoculum

*Metarhizium brunneum* (KVL 12–19) was the strain chosen for this study as part of EU FP7 project INBIOSOIL, representing the same genotype as GranMet/BIPESCO 5. The strain is maintained frozen (−80 °C) at University of Copenhagen, Department of Plant and Environmental Sciences. Stock cultures of the strain were grown on 4% Sabouraud dextrose agar (SDA; Merck, Sweden) in vented Petri dishes and then stored at 8 °C for up to six months. Subcultures were grown by transferring conidia from a stock culture plate onto SDA plates and incubating at 20 ± 1 °C for 20 days. Conidia were harvested by flooding the cultures with sterile 0.05% Triton-X 100 (VWR, Stockholm, Sweden), and scraping with a sterile Drigalski spatula and the resulting suspension was transferred to 50 mL stock tube. Concentrations of the stock suspension were measured in a hemocytometer. To assess conidial viability, germination tests were carried out and viability was determined to be >95%. Stock suspensions of conidia were refrigerated and used the day after preparation.

### 2.3. Soil, Plant Material and Cages

Soil was obtained from the University of Copenhagen experimental farm Bakkegaarden, which has been managed as an organic farm for at least ten years. Pure, sieved soil from the top 20 cm was used in the experiment. Sweet corn (*Zea mays* L.) cv. Sundance, F1 hybrid was used in the experiment.

**Figure 1:**
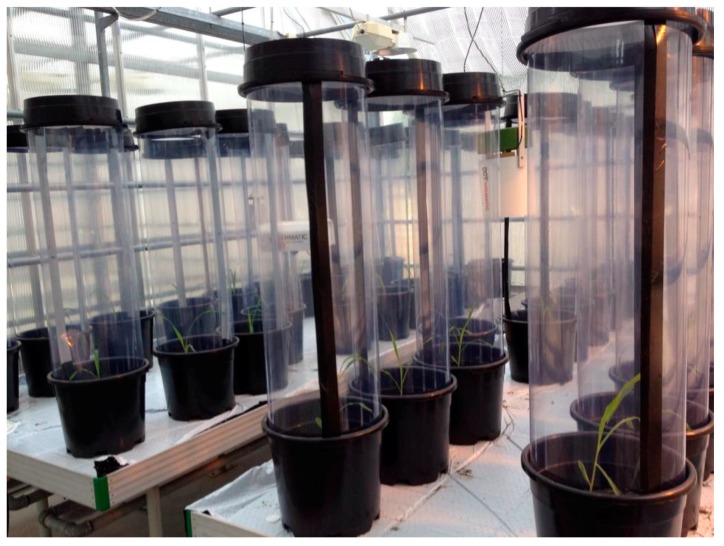
Experimental cages made of plastic Mylar film. Cage tops were covered with fine nylon mesh and secured with plastic rings to prevent insect escape.

Experimental cages (40 cm diameter and 1 m height) were made of plastic Mylar film with the top covered with fine white nylon mesh, firmly fitted on the top of the cage with a plastic ring. In the side of the cage a flap (10 cm × 20 cm) could be opened to facilitate manipulation in the cage. Cages were made so that they fit tightly into the rim of 20 L pots (Figure 1). The pots were filled with soil and depending on treatment, the soil surface (up to 5 cm) was inoculated with 8 × 10^9^ conidia mL^−1^ of *M. brunneum* spore suspension or by adding 0.05% Triton-X 100 on the same day the seeds were sown. Three maize seeds were sown per pot and thinned to one plant seven days after sowing. The thinned plants were used for a separate analysis.

### 2.4. Experimental Design

The experiment was conducted under greenhouse conditions, at 23 ± 2 °C and 15 ± 2 °C, during day and night, respectively, 12 h with light using supplemental light when necessary, and 80%–85% RH. The irrigation was supplied by bottom watering containing a nutrient solution, an ordinary NPK solution with Electrical conductivity 2.00 and pH 6.00. After three weeks when the plants had reached the third foliar stage, they were infested with *R. padi*, first by introducing one barley leaf with five adult aphids onto the maize plant and two days later an additional leaf with 10 adult aphids was added to obtain a sufficient aphid infestation. In treatments with *A. aphidimyza*, twenty pupae were introduced into the pot soil on the same day as aphid infestation and *A. aphidimyza* pupae were positioned equidistantly in 3 cm depth around each plant in a 10 cm perimeter (Table 1).

Cages were placed covering the top of the pots right after *R. padi* were introduced (day 1). The effects of *A. aphidimyza* and *M. brunneum* on *R. padi* were evaluated in a completely randomized block design. Each pot, containing one maize plant infested with *R. padi*, represented one replicate and each treatment was represented by 10 pots, totaling 40 pots (Figure 1). There were four treatments in total: control, fungus only, predator only, and fungus and predator combined.

After the introduction of aphids and *A. aphidimyza* pupae, cages were checked daily. The day of first midge emergence in each cage was noted and the number of midges recorded daily. Aphid count was time consuming so aphids were visually counted in one-fourth of the cages in each treatment daily, without disturbing the plant, thus preventing observers from seeing aphids hidden in the leaf sheets. The experiment was terminated 11 days after the first midge emergence when aphid densities became very low in both predator treatments. For the final assessment, cages were emptied and leaves were examined under the stereomicroscope. This allowed a full count of the number of live aphids and their developmental stage (nymph or adult), consumed aphids, number of *A. aphidimyza* eggs and adults. *Aphidoletes aphidimyza* larvae paralyze the aphid before extracting the body contents [27] leaving the empty aphid fixed to the leaf which makes it possible to count with reasonable precision the aphids with signs of predation.

### 2.5. Presence of the Microbial Inoculum

The presence of *M. brunneum* conidia in the soil was assessed at the end of the experiment by two methods: selective media and insect-bait.

The soil was sampled by removing with a spoon the upper 3 cm of soil from five pots treated with *M. brunneum* and five untreated pots, and then placed individually in plastic bags. A sample of 10 g of soil was mixed with 90 mL of 0.05% Triton-X 100 (VWR, Sweden), diluted to 10^−3^ and plated on selective medium containing 39 g potato dextrose agar (PDA; Sigma-Aldrich, Hamburg, Germany), 1 g yeast, 0.5 g Chloramphenicol, 0.25 g Cycloheximide, 44 µL dodine. The plates were incubated at 23 ± 1 °C for 10 days in the dark, and then the presence of *M. brunneum* was verified.

*Tenebrio molitor* larvae were used as bait insects. Two 155 mL plastic cups were filled with the soil taken from each pot. Ten *T. molitor* larvae were put into each cup; subsequently, the closed cups were incubated at 23 ± 1 °C in the dark and inverted every day. Dead larvae were surface sterilized with 1% Na-Hypochlorite prior to incubation in moist chamber. Visible external growth of fungi and microscopic examination of fungal mycelium provided the basis for fungal identification.

The presence of *M. brunneum* conidia on the leaves was also checked superficially and endophytically. For this assessment, the two weeks old plants thinned in the beginning of the experiment were used: five plants from pots treated with *M. brunneum* and five from untreated pots. The thinned plants were placed in plastic bags, identified by the treatment, and kept in the refrigerator until the next day for evaluation.

The presence of *M. brunneum* on leaves was assessed by printing both sides of a leaf, two leaves per plant, onto selective medium (the same used for the soil). After 3 weeks at 23 ± 1 °C, presence or absence of fungi was confirmed. Endophytic presence of *M. brunneum* was assessed by incubating surface sterilized leaves on selective medium. The leaf surface was sterilized by immersion for 2 min in 0.5% sodium hypochlorite, 2 min in 70% ethanol, rinsed in sterile deionized water three times and dried using sterile filter paper. The outer edges of the leaves were dissected and discarded [28]. The remaining parts were cut into pieces and cultured on selective medium in a Petri plate. Ten plates from each treatment (with and without fungi application) were incubated with three pieces of leaf per plate (two plates per plant). The plates were incubated for three weeks at 23 ± 1 °C.

### 2.6. Molecular Characterization Metarhizium Isolate

The recovered *Metarhizium* strain was firstly morphologically identified and then molecularly identified. DNA was extracted from the conidia harvested from one plate using the DNeasy Plant Mini Kit (QIAGEN, Hilden, Germany) following the manufacturer’s instructions. PCR amplifications were performed for one representative isolate of each multilocus genotype with primers EF2F (5′-GGAGGACAAGACTCACATCAACG-3′) and EFjR (5′-TGYTCNCGRGTYTGNCCRTCYTT-3′) using the conditions described by [29]. PCR products were purified using the GFX PCR DNA and Gel band purification kit (GE Healthcare, Little Chalfont, UK) and sequenced with the same primers. Sequencing was performed by Beckman Coulter Genomics (Essex, UK). The sequence was aligned using GenBank.

### 2.7. Data Analysis

Visual counts of nymphs and adult aphids (log-transformed) were analyzed using a linear mixed-effects model (PROC MIXED) (SAS Inst., Cary, NC, USA, 2008). Main effects were treatment, day, and cage. Cage was set as a random factor, and day as a repeated factor. Full models were reduced by backward removal of non-significant interaction effects. Visual counts of midges, in treatments with the presence of predator, were analyzed using a generalized linear mixed-effects model (PROC GLIMMIX) (SAS Institute, 2008). Main effects were treatment, day and cage. Cage was set as a random factor, day as a repeated factor, and a poisson distribution was selected based on Alkaikes information criterion (AIC). The model was reduced by backward removal of non-significant interaction effects.

The final counts of nymph and adult aphids (log-transformed) and of consumed aphids were also analyzed using a linear mixed-effects model (Proc MIXED) with treatment as main effect. Final counts of midges and eggs (all stages log-transformed) were analyzed using a linear mixed effects model (proc MIXED) with treatment as main effect. Aphid mortality due to treatment was estimated as the difference between treatment and control. Individual treatments were compared using least squares means.

To assess whether the effect of the two treatments *M. brunneum* and *A. aphidimyza* was additive, a Chi-square analysis was conducted comparing aphid mortality in fungus and predator combined treatment to the sum of aphid mortality in fungus only and predator only treatments.

## 3. Results

### 3.1. Presence of The Microbial Inoculum

The presence of *M. brunneum* was confirmed in the soil samples from fungal treated pots. Baited *T. molitor* larvae yielded *Metarhizium* spp. infections in 64% of the larvae from sampled cups, whereas no larvae showed signs of infection in the control. Also, by selective medium, the presence of *M. brunneum* was confirmed in all soil samples from fungal treated pots. Fungal colonies were not observed on plates from the control.

The presence of *M. brunneum* was confirmed on the surface of 60% of printed leaves from plants grown in the fungal treated pots. As expected, the fungus was not isolated on the leaves from the control. No endophytic association was found.

The aligning of the 5′ EF1-α sequence, obtained from *Metarhizium* isolated on the surface of the maize leaves, with BLAST identified it as *Metarhizium brunneum*.

### 3.2. Impact of M. brunneum on A. aphidimyza

**Figure 2:**
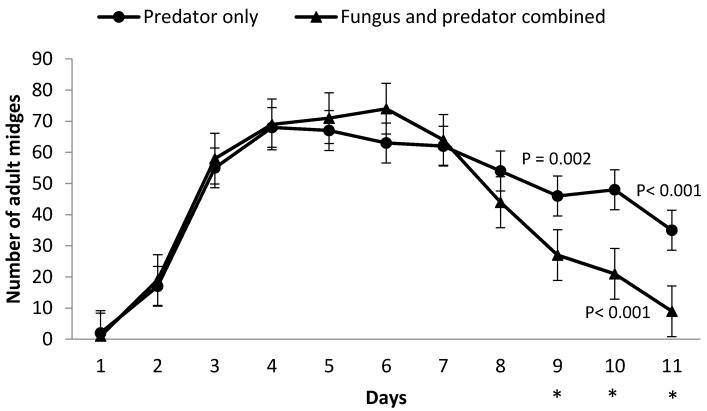
Daily numbers of *A. aphidimyza* midges in the absence (predator only treatment) or presence (fungus and predator combined treatment) of *M. brunneum* from first day of emergence until last day of experiment. Asterisks indicate statistically significant (*p* < 0.05) differences among treatments.

The emergence of *A. aphidimyza* midges began on the fourth day after the pupae were introduced into the cages—day 4 after aphid introduction. In both treatments with *A. aphidimyza*, midge emergence started on the same day and the numbers of adult midges were not significantly different from the first to the eighth day between both treatments. The numbers of *A. aphidimyza* midges peaked on the fourth day in the predator only treatment and on the 6th day in the fungus and predator combined treatment (Figure 2). There was a significant interaction effect of treatment × day on midge numbers (*F*_10,180_ = 10.39, *p* < 0.0001). Pairwise comparisons showed significant differences between the treatments predator only and fungus and predator combined on the last three days of the study, ninth (*t* = 2.84, *p* = 0.006, df = 82.02), 10th (*t* = 4.19, *p* < 0.0001, df = 88.2) and 11th (*t* = 5.17, *p* < 0.0001, df = 90.8) days after pupae had been introduced to the cages (Figure 2). There was no significant effect of treatment on the number of eggs laid by *A. aphidimyza* female midges (*F*_1,18_ = 0.33, *p* < 0.573), indicating that the entomopathogen *M. brunneum* had no significant effect on oviposition.

### 3.3. Impact of A. aphidimyza and M. brunneum on R. padi

The mixed linear model showed a significant effect of treatment × day on number of aphids (log-transformed) (*F*_21,84_ = 2.94, *p* = 0.0002). The total number of aphids at the final count also showed a significant effect of treatment (*F*_3,36_ = 15.75, *p* < 0.0001). There were significant effects of *M. brunneum* and *A. aphidimyza*, separately, on the *R. padi* population (χ^2^ = 56.07, df = 1, *p* < 0.0001, χ^2^ = 537.76, df = 1, *p* < 0.0001, respectively).

The fungus + predator combined treatment suppressed the *R. padi* population significantly compared to the control (*t* = 4.14, *p* = 0.0002, df = 36 and *t* = 6.53, *p* < 0.0001, df = 36, nymphs and adults respectively).

**Figure 3:**
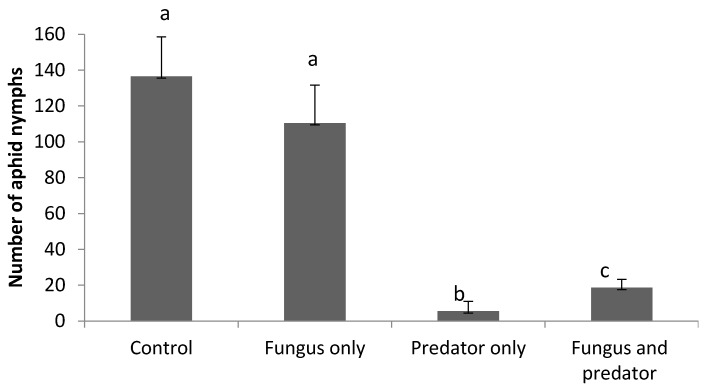
Comparison of the numbers (±SE) of living *R. padi* nymphs at the end of the study between treatments treated or untreated with *A. aphidimyza* (*n* = 40) and *M. brunneum*. Letters above columns indicate significant (*p* < 0.05) differences among treatments.

*Rhopalosiphum padi* nymph population was significantly affected by treatment (*F*_3,36_ = 26.36, *p* < 0.0001). Pairwise comparisons showed no significant differences between the control and fungus-only treatment on the *R. padi* population. (*t* = 1.22, *p* = 0.280, df = 36). However, *A. aphidimyza* suppressed *R. padi* nymphs significantly more than the combined predator + fungi treatment. (*t* = −3.97, *p* = 0.0003, df = 36) (Figure 3).

**Figure 4:**
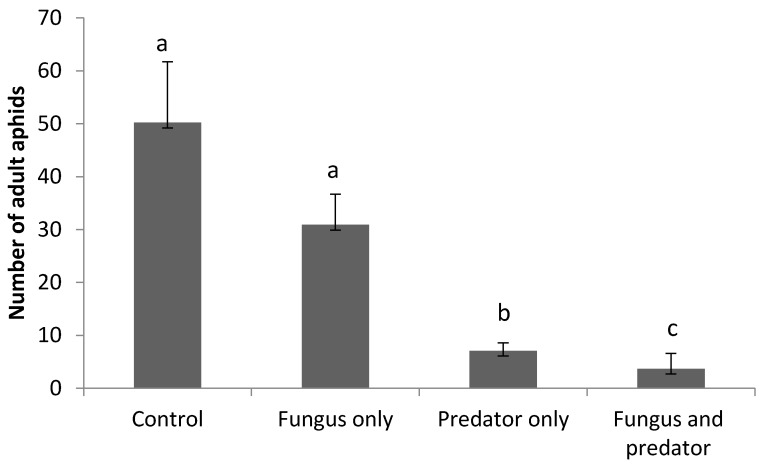
Comparison of the numbers (±SE) of living *R. padi* adults at the end of the study between treatments treated or untreated with *A. aphidimyza* (*n* = 40) and *M. brunneum*. Letters above columns indicate significant (*p* < 0.05) differences among treatments.

*R. padi* adults were significantly influenced by treatment (*F*_3,36_ = 17.21, *p* < 0.0001). Pairwise comparisons showed no significant differences in the *R. padi* adult population between the control and fungus-only treatment. (*t* = 1.22, *p* = 0.230, df = 36). However, in contrast to *R. padi* nymphs, the adults were suppressed most in the fungus + predator combined treatment rather than the predator-only treatment. (*t* = 2.42, *p* = 0.020, df = 36) (Figure 4). When comparing the fungus-only and predator-only treatments, *R. padi* nymphs and adults were clearly suppressed most by *A. aphidimyza* alone. (t = 7.01, *p* < 0.0001, df = 36 and *t* = 2.89, *p* = 0.006, df = 36, respectively) (Figure 3 and Figure 4). Also, the number of consumed aphids was significantly higher in the predator only treatment than in the fungus and predator combined treatment at the final assessment (*t* = 3.65, *p* = 0.002, df = 18).

The suppression of the aphid population found in the fungus and predator combined treatment was significantly less than the sum of aphid populations from predator only and fungus and predator combined treatments, with the natural enemies applied separately (χ^2^= 983.8, df = 1, *p* < 0.0001).

## 4. Discussion

It is well known that entomopathogenic fungi are effective microbiological control agents; however, it is essential to ensure that they do not negatively affect non-target organisms, such as beneficial insects. The present study examines, innovatively, the potential impact of an entomopathogenic fungus, *M. brunneum*, applied directly into the soil, on the plant-dwelling aphid *R. padi* and on the soil-dwelling pupal, adult, egg, and larval stages of the predator *A. aphidimyza* under greenhouse conditions. The experimental setting simulated a situation in which *A. aphidimyza* pupae were exposed to soil with high fungal concentration, newly hatched adults emerged through the soil to lay eggs on maize plants infested by aphids; therefore, this greenhouse study represents a multitrophic plant-fungus-insect context.

The observed reduction in adult aphid population in the *M. brunneum* treatment may have been caused by fungal presence on the surface of the maize leaves. The presence of *M. brunneum* conidia on the surface the leaves was confirmed and it may have occurred during plant growth through the soil or by transportation from soil to leaves by insects. According to [30], aphids are able to distribute entomopathogenic fungi from soil to leaves, this is an evidence of the plant- or insect- mediated interactions between fungi in soil and plant-living insects. It is well known that direct fungal application can affect the aphid survival rates and the number of offspring produced per aphid female per day [31,32], while no other studies investigated of soil applied *M. brunneum*.

Diversity of natural enemies improves biological control of a shared pest if they are complementary [33]. Effects of *Beauveria bassiana* on parasitoids of the green peach aphid, *Aphidius matricariae* and *Aphidius* colemani, have been investigated respectively by [34,35]. The authors found, in both studies, that with appropriate timing, the parasitoids and *B. bassiana* could be combined for the biological control of *M. persicae*. These studies showed that the combination of biological control agents requires effective time management to avoid antagonistic interactions. We obtained better control in the combined treatment, but the effect was less than additive, pointing to an antagonistic effect of *M. brunneum* on *A. aphidimyza*. Also, the suppression of the aphid population was significantly less in the fungus and predator combined treatment than the sum of aphid populations from predator only and fungus and predator combined treatments. Therefore, use of *M. brunneum* requires consideration of both timing and method of application to protect non-target predators.

It was hypothesized that *A. aphidimyza* would be exposed to a pathogen on two occasions, during its pupal stage in the soil, especially when the young adult emerged, and again when the adult and its offspring came into contact with the plant/aphids. As expected, the initial number of *A. aphidimyza* adult midges emerging was similar in control and *M. brunneum*-treated cages, suggesting that the fungus did not readily infect the predator in its pupal stage. *Aphidoletes aphidimyza* pupae are covered by a cocoon structure which impedes natural enemy attacks and provides anti-bacterial and anti-fungal protection [36].

Though the number of adult midges in the predator only treatment peaked two days before the fungus and predator combined treatment, the number of midges in the fungus and predator combined treatment began to decline before then predator only treatment and on the last day of experiment, the predator only treatment had four times more midges than the fungus and predator combined treatment. The suppressed final total number of midges, as well as the later peak and earlier decline of midge numbers in the fungus and predator combined treatment may represent effects of the presence of *M. brunneum* in this system. Considering that *A. aphidimyza* midges have a life span of approximately seven days and most males emerge before females [37], a decrease in male longevity can be critical for successful mating and can thus directly affect reproduction. *Culicoides* biting midges (Diptera: Ceratopogonidae), vectors of several arboviruses, showed high larval mortality when exposed directly to *M. anisopliae* [38]. According to [39], all *Culicoides* biting midges were found to be infected by *M. anisopliea* four days after being exposure to a tissue paper dusted with “dry” conidia of the fungi. *Aphidoletes aphidimyza* midgelongevity can be affected by the content of the honeydew produced by different aphid species and female longevity affects lifetime fecundity [40]. However, little is known about *A. aphidimyza* longevity and the factors that can affect it, especially when combined with fungal pathogens.

The higher number of aphid nymphs in the fungus and predator combined treatment and higher number of aphids consumed in predator only treatment can be considered a consequence of the delay in *A. aphidimyza* life cycle (or emergence) in the fungus and predator combined treatment due to the *M. brunneum* presence. It was assumed that in the predator only treatment egg laying commenced earlier than in the fungus and predator combined treatment. The *A. aphidimyza* predation rate can be affected by other external factors such as the presence of a generalist predator and different N fertilization levels as showed by [41].

There is a lack of studies combining *A. aphidimyza* with fungal pathogens. According to [42], *A. aphidimyza* is compatible with the entomopathogenic fungi *Lecanicillium logisporum*. However, the report includes no methodological or experimental information.

In a wider perspective, the application of entomopathogenic fungi brings many benefits to maize crops such as controlling *D. virgifera*, a root damaging pest. Considering the situation where, besides aphid pest, other pests are attacking the plant, such as *D. virgifera* that causes significant high mortality [43], the application of the entomopathogenic fungi would be encouraged even though some negative side-effect of the fungus on *A. aphidimyza* and other naturally occurring or mass-released predators would be expected, as long as the biocontrol of aphids was still achieved, or even with less effect on aphids if the soil living pest was the major cause of losses.

## 5. Conclusions

Based on the results of this study, *A. aphidimyza* predation is more effective than *M. brunneum* in suppressing and controlling *R. padi* population on maize plants. Combining *A. aphidimyza* and *M. brunneum* showed an effect on *R. padi*, though the *M. brunneum* was applied to the soil. The combined effect on *R. padi* was less than additive, and an earlier decline in the number of adult midges was a negative side-effects on *A. aphidimyza*. For biocontrol purposes, the side-effects of soil application of *M. brunneum* on the performance of *A. aphidimyza* can be considered minor.

## Figures and Tables

**Table 1:** The number and date of release of aphids and biological control agents over the course of the experiment.

Organism	Release Date	Corresponding Week	Number of Organisms Released
*M. brunneum* (suspension)	18 March 2015	0	8 × 10^9^ conidia/pot
*R. padi* (adults)	10 April 2015	3	5/pot
	13 April 2015	4	10/pot
*A. aphidimyza* (pupae)	16 April 2015	4	20/pot
